# TGRL Lipolysis Products Induce Stress Protein ATF3 via the TGF-β Receptor Pathway in Human Aortic Endothelial Cells

**DOI:** 10.1371/journal.pone.0145523

**Published:** 2015-12-28

**Authors:** Larissa Eiselein, Tun Nyunt, Michael W. Lamé, Kit F. Ng, Dennis W. Wilson, John C. Rutledge, Hnin H. Aung

**Affiliations:** 1 Department of Internal Medicine, Division of Cardiovascular Medicine, School of Medicine, University of California Davis, Davis, California, 95616, United States of America; 2 Department of Molecular Biosciences, School of Veterinary Medicine, University of California Davis, Davis, California, 95616, United States of America; 3 Department of Pathology, Microbiology, and Immunology, School of Veterinary Medicine, University of California Davis, Davis, California, 95616, United States of America; Georgia Regents University, UNITED STATES

## Abstract

Studies have suggested a link between the transforming growth factor beta 1 (TGF-β1) signaling cascade and the stress-inducible activating transcription factor 3 (ATF3). We have demonstrated that triglyceride-rich lipoproteins (TGRL) lipolysis products activate MAP kinase stress associated JNK/c-Jun pathways resulting in up-regulation of ATF3, pro-inflammatory genes and induction of apoptosis in human aortic endothelial cells. Here we demonstrate increased release of active TGF-β at 15 min, phosphorylation of Smad2 and translocation of co-Smad4 from cytosol to nucleus after a 1.5 h treatment with lipolysis products. Activation and translocation of Smad2 and 4 was blocked by addition of SB431542 (10 μM), a specific inhibitor of TGF-β-activin receptor ALKs 4, 5, 7. Both ALK receptor inhibition and anti TGF-β1 antibody prevented lipolysis product induced up-regulation of ATF3 mRNA and protein. ALK inhibition prevented lipolysis product-induced nuclear accumulation of ATF3. ALKs 4, 5, 7 inhibition also prevented phosphorylation of c-Jun and TGRL lipolysis product-induced p53 and caspase-3 protein expression. These findings demonstrate that TGRL lipolysis products cause release of active TGF-β and lipolysis product-induced apoptosis is dependent on TGF-β signaling. Furthermore, signaling through the stress associated JNK/c-Jun pathway is dependent on TGF-β signaling suggesting that TGF-β signaling is necessary for nuclear accumulation of the ATF3/cJun transcription complex and induction of pro-inflammatory responses.

## Introduction

Although the exact regulatory mechanisms are not yet understood, many studies have found increased apoptotic signals in vascular cells of atherosclerotic plaques compared to normal vessels [1–4]. Well-known risk factors for the development of atherosclerosis, such as high glucose levels [5], increased oxidative stress, angiotensin II [6] and oxidized low-density lipoprotein (LDL) [7] have all been shown to induce apoptosis in endothelial cells.

Triglyceride-rich lipoproteins (TGRL) include chylomicrons, which contain triglycerides derived from the exogenous (intestine-derived) pathway, and VLDL (very low-density lipoprotein), which contain triglycerides from the endogenous (liver-derived) pathway. Lipolysis of TGRL occurs when lipoproteins bind to lipoprotein lipase (LpL) [8], an enzyme anchored to the surface of endothelial cells [9, 10]. While cultured endothelial cells lack surface LpL, we have modeled the effects of TGRL lipolysis using pre-treatment of postprandial lipids isolated from humans with exogenous LpL. TGRL lipolysis releases neutral and oxidized free fatty acids (FFAs) that induce endothelial cell inflammation in high physiological and pathophysiological concentrations and injure endothelial cells by increasing VLDL remnant deposition in the artery wall [11, 12], augment endothelial monolayer permeability, perturb zonula occludens-1 and F-actin, and induce apoptosis [13]. TGRL lipolysis products also significantly increase the production of reactive oxygen species (ROS) in endothelial cells and alter lipid raft morphology [11]. Pro-inflammatory pathways are activated and predominate when endothelial cells are exposed to high physiological and pathophysiological concentrations of TGRL lipolysis products, but not TGRL alone [14–16]. We recently reported that TGRL lipolysis products activate stress response pathways that induce expression of multiple pro-inflammatory and pro-apoptotic genes leading to endothelial dysfunction [17]. We identified transcription of ATF3, a member of the CREB family, as a key response gene after treatment with TGRL lipolysis products and demonstrated that its induction was essential for the expression of a subset of pro-inflammatory responses. The signaling mechanisms behind these phenomena, however, are not well understood.

Activating transcription factor 3 (ATF3) is both constitutively expressed and induced by a variety of signaling pathways. ATF3 binds to a variety of AP-1 sequence promoter sites as a homodimer or as a heterodimer with other members of the AP-1 transcription factor family such as c-Jun [18]. ATF3 appears to play a complex central role in modulating pro-inflammatory responses as ATF3 deficient mice are more susceptible to sepsis [19] and ventilator induced lung injury [20]. Further, a variety of stress-stimuli, which are also known risk factors for the development of atherosclerosis, can rapidly induce ATF3 in endothelial cells. These include ox LDL, homocysteine, TNF-α or palmitate [21]. Our findings demonstrated that ATF3 induction by products of TGRL lipolysis was dependent on the stress response MAP kinase pathway acting through JNK and c-Jun [17].

TGF-β1 is released from a secreted latent complex and is involved in many different biological processes, such as cell growth, cell cycle progression, migration, differentiation, matrix production and apoptosis [22, 23]. Studies have shown that in vascular endothelial cells, TGF-β1 is a potent inducer of apoptosis [24, 25]. TGF-β1 signaling occurs when specific type I and type II serine/threonine kinase receptors form heteromeric complexes [26]. The type I receptor, also termed activin receptor-like kinase (ALK), is then phosphorylated by the constitutively active TGF-β type II receptor (TβR-II). Although several ALKs and type II receptors exist in vertebrates, the typical receptor combination for TGF-β1 signaling is ALK-5/TβR-II. Second messenger activation occurs when phosphorylated ALK in turn phosphorylates receptor-regulated Smads (R-Smads). Phosphorylation of Smad2 and Smad3 proteins results in dissociation from the receptor and binding to the common mediator Smad4. The Smad-complex then translocates to the nucleus where it regulates transcription of target genes by interacting with many specific DNA-binding proteins.

Several studies have shown that the TGFβ-1/Smad cascade is closely linked to the p53 signaling network [27–29] and that TGFβ-1 can increase p53 expression in various cell types [30]. p53 protein is a transcription factor that regulates the expression of a wide variety of genes involved in cell cycle arrest and apoptosis in response to genotoxic or cellular stress. Our previous study [17] and others [31, 32] had shown that JNK activates ATF3 through transcriptional regulation. JNK activation also results in phosphorylation of c-Jun which in turn can form a complex with ATF3 with subsequent binding to AP-1 responsive promoter regions. While both ATF3 and c-Jun promote apoptosis [33], this is opposed by JunB, another transcription factor activated through the NFκB pathway.

Oxidized LDL, a mediator for the development of atherosclerotic cardiovascular disease, has been demonstrated to activate the TGFβ-1/Smad signaling cascade in glomerular mesangial cells [34, 35] and is known to be a potent inducer of apoptosis in endothelium [7, 36–38]. Like oxidized LDL, lipolysis products generated from the hydrolysis of triglyceride-rich lipoproteins (TGRL), are now increasingly appreciated as active participants in the development of atherosclerosis.

Recent studies have suggested that ATF3 up-regulation and activity as a transcription factor can be controlled by, and depends on, prior TGFβ receptor activation. In epithelial cells, TGF-β stimulation has been shown to rapidly induce the expression of ATF3 via a Smad3 containing transcriptional complex [39]. Furthermore, ATF3, in turn, has been shown to directly interact and complex with Smad3 and Smad4 to act as a transcriptional repressor [39]. Our prior studies demonstrating TGRL lipolysis products induce endothelial cell apoptosis suggested a potential role for TGF-β in the response. Here, we test the hypothesis that the observed lipolysis product-induced up-regulation and activation of ATF3 in HAEC is regulated by the TGF-β signaling system.

## Materials and Methods

### Human TGRL isolation

The protocol for obtaining human TGRL (Protocol No. 223062) was approved by the Human Subjects Review Committee/IRB at the University of California Davis. The participants were written informed consent to participate in this study. The informed consent was also approved by the ethics committees/IRB. Postprandial blood samples were obtained 3.5 h after consumption of a moderately high fat meal, which corresponds to the peak elevation in plasma triglyceride concentrations. TGRL were isolated from human plasma at a density of less than 1.0063 g/mL following an 18 h centrifugation at 40,000 rpm in a SW41 Ti swinging bucket rotor (Beckman Coulter, Sunnyvale, CA) held at 14°C within a Beckman L8-70M (Beckman) ultracentrifuge. The top fraction TGRL was collected and dialyzed in Spectrapor membrane tubing (mol wt cut off 3,500; Spectrum Medical Industries, Los Angeles, CA) at 4°C overnight against a saline solution containing 0.01%EDTA. The TGRL lipolysis mix routinely used in experiments was normalized based on triglyceride concentrations and was diluted to contain 150 mg/dL of triglycerides. Total triglyceride content of samples was determined using the serum triglyceride determination kit (Sigma Aldrich cat # TR0100). The kit converts triglycerides to free fatty acids and glycerol and glycerol is assayed enzymatically.

### Reagents and antibodies

Lipoprotein lipase (LpL) (L2254), monoclonal anti-β-actin antibody (A 5441), and an inhibitor of activin receptor-like kinases (ALKs 4, 5, and 7), SB 431542 (ALK) were purchased from Sigma, St. Louis, MO. Human TGF-β1 (100B) and recombinant human TGF-β1 (anti TGF-β1 antibody) (MAB240) from R&D Systems Inc., Minneapolis, MN. Antibodies were purchased from the following sources: phospho-Smad2/3 (Ser423/425) (sc-11769-R), Smad4 (sc-7154), ATF3 (sc-188), c-Jun (sc-1694), p53 (sc-6243) and CD-36 (sc-5523) from Santa Cruz Biotechnology, Santa Cruz, CA; Smad2 (#3103), p-Smad2 (Ser465/467)/Smad3 (Ser423/425) (#8828)and p-c-Jun (#9261) from Cell Signaling Technology (Danvers, MA); caspase-3 (IMG-144A) from (Imgenex, San Diego, CA); Nuclear Loading Control Anti-TATA binding protein TBP antibody (ab63766) from Abcam (Cambridge, MA); HRP-conjugated secondary anti-mouse (Amersham # NXA931) or anti-rabbit antibody (Amersham #NA9340V) from GE-Biosciences. Isotype control for rabbit IgG (AB-105-C) and mouse IgG (MAB-002) were purchased from R & D system, Minneapolis, MN. Secondary Goat anti-rabbit antibody conjugated to Alexa fluor 488 (A-11034) was purchased from Molecular Probes, Eugene, OR.

### Cell culture and lipid treatments

Human aortic endothelial cells (HAEC) (passage 6, Cascade Biologics, Portland, OR) were cultured in EBM-2 basal media (cc-3156) supplemented with EGM-2 SingleQuot Kit (cc-4176) (Lonza, Walkersville MD) under and atmosphere of 5% CO_2_: 95% air at 37°C. Cells were exposed to the following conditions, media, TGRL (150 mg/dL = 1.5 mg/mL), lipoprotein lipase (LpL) (2 U/mL), and TGRL lipolysis product (TGRL (150 mg/dL) + LpL (2 U/mL)). The final concentration of TGRL, LpL, and TGRL lipolysis products were diluted in media and pre-incubated for 30 minute at 37°C prior to application. For positive control, cells were treated with 20 ng/mL of human TGF-β1 for 3 h. To study the involvement of TGF-β signaling, following co-incubation experiments were performed. ALK, SB 431542 (10 μM) and 4 μg/mL of anti TGF-β1 antibody were co-incubated with HAEC and TGRL lipolysis products for 3 h. To test the CD36 receptor biding to FFAs from TGRL lipolysis products, CD36 receptor blocking antibody (2 μg/mL) was co-incubated with TGRL lipolysis products for 3 h. After incubation, cells were washed with cold PBS and harvested based on experimental endpoints.

mRNA and protein expression were analyzed at the following time points: TGF-β1 release at 15 min, 30 min and 45 min; p-Smad2/3 and Smad2 protein expression at 1.5 h; Smad4 translocation at 1, 2 and 3 h; p53 protein expression at 0.5, 1.5 and 3 h; ATF3, p-c-Jun, caspase-3 protein expression and mRNA expression of ATF3, IL-8, E-selectin, JunB, IL-6, NFKB1 (NFκB) and NFKBIA (IκBA) at 3 h.

### TGF-β1 Immunoassay

HAEC were grown to confluence in 6-well medical grade polystyrene plates (BD Falcon, Franklin Lakes, NJ) and were then stimulated with media control (M) or TGRL (T, 150 mg/dL) alone, LpL (L, 2 U/mL) alone or TGRL lipolysis products (TL, TGRL plus LpL) for 15 min, 30 min and 45 min. Cell culture supernatants and lysate were collected and kept at -80C°. Human TGF-β1 protein expression was analyzed using enzyme-linked immunosorbent assay for quantitative detection of Human/Mouse TGF-β1 ELISA Ready-SET-Go! kit (#88–8350) from eBioscience, Inc. (San Diego, CA) according to manufacturer’s instructions. Microplates were scanned at a wavelength of 450 nm using a SpectraMax plate reader (Molecular Devices, Sunnyvale, CA). Supernatant concentration of activated TGF-β1 was evaluated following the manufacturers’ instructions based on standard curves. Each experiment was performed at least four times.

### Western blotting

To study TGF-β/ATF3 signaling pathways, we performed Western Blot analysis. HAEC were grown to confluence in T-75 cell culture flasks and then treated as described above for 0.5, 1.5 or 3 h. After the incubation, cells were washed twice with PBS (without Ca and Mg), scraped with cold PBS and centrifuged at 3,000 rpm for 10 min at 4°C. Cell pellets were lysed in radioimmune precipitation assay (RIPA) buffer containing 50 mM Tris (pH 7.4), 150 mM NaCl, 1% NP40, 0.25% sodium deoxycholate, 0.1% SDS, 1x Protease inhibitor cocktail set 1 (Calbiochem, La Jolla CA), 1mM NaF, and 1mM Na_3_VO_4_. Protein concentration was determined with the bichinoic acid assay (Pierce), and equal amounts of proteins (50 μg) were separated by SDS-PAGE. The 11% total acrylamide, 2.75% crosslinker running gel was overlaid with a 4% total acrylamide, 2.75% crosslinker stacking gel and run at 4°C on a Hoefer SE-600 vertical tank chamber (Hoefer Scientific Instruments, San Francisco, CA) at 20 mA for an average of 5.5 h. Proteins were then transferred onto 0.2-μm polyvinylidene difluoride membranes (Bio-Rad, Hercules, CA) which were subsequently blocked with 5% nonfat milk (Bio-Rad) for 1 h and then probed with either Smad2 (1:1000), p-Smad2/3 (1:200), Smad4 (1:200), p53 (1:200), ATF3 (1:200), c-Jun (1:200), p-c-Jun (1:1000), caspase-3 (3 μg/mL) or blotting control mouse monoclonal anti-β-actin (1:5,000) or nuclear loading control rabbit polyclonal anti-TBP (2 μg/mL) at 4°C overnight. Membranes were then incubated with HRP-conjugated secondary anti-mouse or anti-rabbit antibody (1:5,000) for 1 h. Blots were developed with the enhanced chemiluminescence detection system according to manufacturer's instructions (Amersham). Protein expression levels were determined using a densitometer and Image Quant.

### Immunofluorescence analysis

The cellular localization of endogenous as well as transiently expressed Smad4 and ATF3 was analyzed by fluorescence microscopy. HAEC were grown to confluence on fibronectin-coated 12-mm round coverslips placed in 24-well medical-grade polystyrene plates (BD Falcon) and were treated (n = 6 coverslips per treatment group) as described above. After treatment, cells were fixed with 4% paraformaldehyde in PBS for 30 min at room temperature and then washed 3X with PBS. Cells then were permeabilized and blocked with Superblock (Pierce, Rockford, IL) containing 0.05% saponin for 30 min. Fixed cells were incubated with rabbit polyclonal anti-phospho-Smad2 (1:200 dilution) or Smad4 (1:100 dilution) or ATF3 (1:100 dilution) overnight and washed with 4X 10% superblock with 0.05% saponin to remove unbound antibody. Subsequently cells were treated with goat anti-rabbit antibody conjugated to Alexa fluor 488 (2 μg/mL) for 1 h, unbound material was removed as above. The nucleus was counterstained for 5 min with 4', 6-diamidino-2-phenylindole (DAPI) (1 μg/mL). Isotype controls were correspondingly run for all primary antibodies used in the study. After cells were mounted, they were evaluated and photographed with an Olympus BX61 system fluorescence microscope; but, p-Smad2 which was evaluated with Delta Vision Deconvolution Microscopy. The images shown are representative of at least six separate experiments. To assess the degree of nuclear accumulation of either Smad4 or ATF3, cells from five random view frames for six coverslips in each treatment group were counted as showing either fluorescing or non-fluorescing nuclei. Percent translocation was calculated as cells with green fluorescing nuclei divided by the total number of cells counted.

### mRNA expression by quantitative RT-PCR (qRT-PCR)

Total RNA was extracted from cells in each of four T-25 flasks/per treatment group (Media, LpL, TGRL, and TGRL lipolysis products) using an RNeasy Mini Kit (Qiagen, Valencia, CA) including the DNA digestion step as described by the manufacturer. An aliquot equivalent to 5 μg of total RNA extracted from each sample was reverse-transcribed to obtain cDNA in a final volume of 21 μL using SuperScript III First-Strand Synthesis System (Invitrogen, Carlsbad, CA). The real-time polymerase chain reaction (RT-PCR) with SYBR Green as fluorescent reporter was used to quantify the gene expression. All the gene specific primers (Table 1) were designed with Primer Express 1.0 software (Applied Biosystems) using the gene sequences obtained from previous data Affymetrix Probeset IDs [17]. Reactions were carried out in 384-well optical plates containing 25 ng RNA in each well. The quantity of applied RNA was normalized by simultaneously amplifying cDNA samples with glyceraldehyde-3-phosphate dehydrogenase (GAPDH)-specific primers. The transcript levels were measured by real-time RT-PCR using the ABI ViiA^™^7 Real-Time PCR system (PE Applied Biosystems, Foster City, CA). The PCR amplification parameters were: initial denaturation step at 95°C for 10 min followed by 40 cycles, each at 95°C for 15 s (melting) and 60°C for 1 min (annealing and extension). A comparative C_T_ method [40] was used to calculate relative changes in gene expression determined from real-time quantitative PCR experiments (Applied Biosystems User Bulletin No.2 (P/N4303859). The threshold cycle, C_t_, which correlates inversely with the target mRNA levels, was measured as the cycle number at which the SYBR Green emission increases above a preset threshold level. The specific mRNA transcripts were expressed as fold difference in the expression of the specific mRNAs in RNA samples from the TGRL lipolysis product-treated cells compared to those from the control-treated cells.

**Table 1:** Oligonucleotide sequence for each primer.

Gene	Primer sequence (5' - 3')
**GAPDH**	Sense-CACCAACTGCTTAGCACCCC
	Antisense-TGGTCATGAGTCCTTCCACG
**ATF3**	Sense-TTCTCCCAGCGTTAACACAAAA
	Antisense-AGAGGACCTGCCATCATGCT
**E-selectin**	Sense-TGGCAATGAAAAATTCTCAGTCA
	Antisense-TCAAGGCTAGAGCAGCTTTGG
**IL8**	Sense-CCTTTCCACCCCAAATTTATCA
	Antisense-TGGTCCACTCTCAATCACTCTCAG
**IL6**	Sense-CTGCGCAGCTTTAAGGAGTTC
	Antisense-TTGCCGAAGAGCCCTCAG
**NFkBIA (IκBA)**	Sense-CTTTTGGTGTCCTTGGGTGC
	Antisense-GCCATTACAGGGCTCCTGAG
**NFKB1 (NFκB)**	Sense-GTCAGAGAGCTGGTGGAGGC
	Antisense-AATTGCTTCGGTGTAGCCCA
**JunB**	Sense-AATGGAACAGCCCTTCTTACCACGA
	Antisense-GGCTCGGTTTCAGGAGTTTGTAGT

### TUNEL Apoptosis assay

To determine TGRL lipolysis induced apoptosis in endothelium of intact mouse carotid arteries, deeply anesthetized mice were perfused with T or TL through the left ventricle for 15 minutes and TUNEL assay was performed on paraffin embedded sections of carotid arteries. All experiments, IACUC protocol #15946, were approved by the Institutional Animal Care and Use Committee of the University of California, Davis. Animals were housed in a temperature and humidity controlled environment with a 12 hour light/dark cycle in the UC Davis Mouse Biology Program. For these studies, C57BL/6 mice were anesthetized with pentobarbital and a cannula was placed in the left ventricle of the heart. Perfusion was performed (N = 4 mice per group) with Media, TGRL (T), or TGRL lipolysis products (TL) at 1.5–2 mL per min for 15 min. Perfusion solutions were administered with 95% O_2_, 5% CO_2_. Tissues were then fixed by perfusion of 10% neutral buffered formalin for 15 minutes and sections of cervical musculature containing carotid arteries fixed overnight and embedded in paraffin. Paraffin sections (5 μm) were deparaffinized and rehydrated. TUNEL reaction was performed using DeadEndTM Fluorometric TUNEL system from Promega (Madison, WI) as described by the manufacturer. Tissues were counterstained with DAPI and cover slipped with Prolong gold. Cross sections of both internal and external carotid arteries from three levels of the cervical musculature were imaged with an Olympus BX61 fluorescence microscope. Photoshop CS was used to identify nuclei with enhanced fluorescein staining. A color range was selected using the eyedropper tool. When the bright green pixels associated with fluorescein-positive nuclei are selected, all nuclei containing this color range are automatically highlighted and are then counted manually. The images shown are representative of three separate experiments.

#### Statistical Analysis

Data for changes in gene expression obtained by qRT-PCR and protein expression were analyzed by GraphPad PRISM software (San Diego, CA). An unpaired student’s t test or two-way analysis of variance with repeated measures was used for comparisons between treatments. Differences with *P*≤ 0.05 were considered significant. Results are expressed as MEAN ± SEM.

## Results

### TGRL Lipolysis Products Stimulate TGF-β1 Release

**Fig 1:**
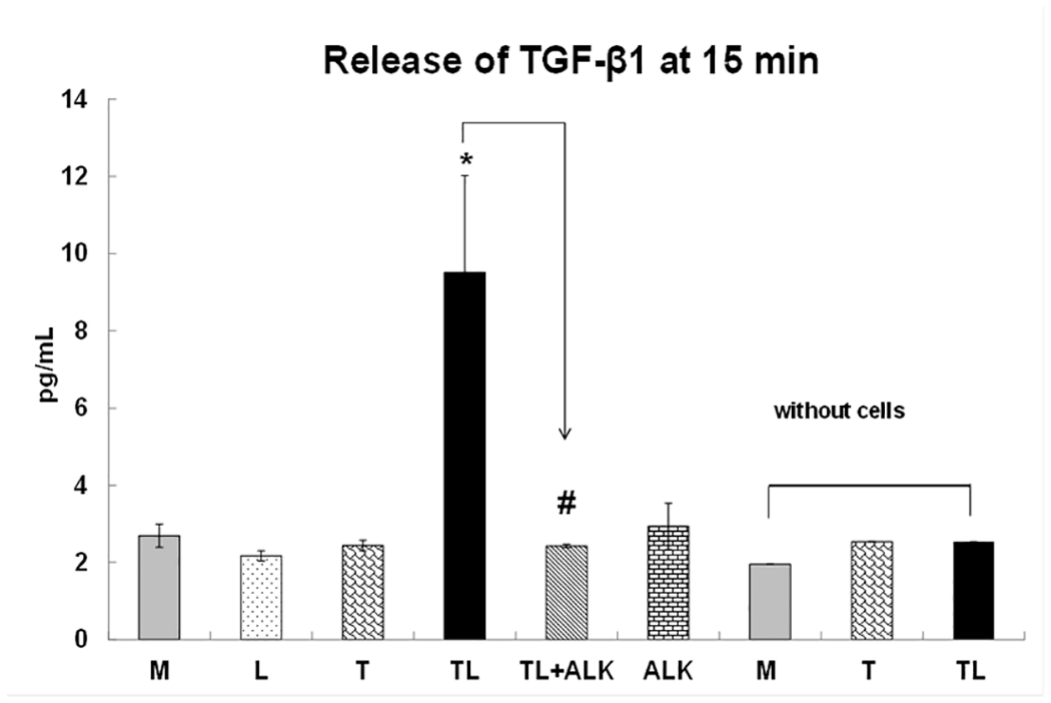
TGRL lipolysis products release TGF-β1 at 15 min. The rate of TGF-β1 release is significantly increased for cells treated with TGRL (150 mg/dL) + LpL (2 U/mL) (TL) compared to cells treated with Media (M) or LpL alone (L) or TGRL alone (T), at 15 min. Addition of 10 μM of ALK to TL (TL+ALK) suppressed TGF-β1 released by TL. N = 4/treatment group, *P*≤0.05 as significant, * = TL compared to M, L, T or TL, # = TL+ALK compared to TL. TGF-β1 was not detected in M, T or TL only, in the absence of cells.

To determine whether TGF-β1 release could be stimulated by TGRL lipolysis products, we analyzed both cell culture supernatants and cell lysates for TGF-β1 by ELISA. The rate of release of TGF-β1 into cell culture supernatants was significantly increased for TGRL lipolysis products (TL) treatments relative to media (M), or LpL (L) or TGRL (T) alone at 15 min (Fig 1). The time course of TGF-β1 release was short as no increase was evident release at 30 min, 45 min in S1A and S1B Fig. In addition, treatment with 10 μM TGF-β inhibitor (ALK) significantly decreased TGF-β1 release. TGF-β1 concentrations in the supernatant of cells treated with media trended higher than that in media without cells. TGF-β1 concentrations in cell free incubations of T, or TL were similar to that in cells treated with media alone. Cell treatment with M, L, T or ALK only slightly increased TGF-β1 compared to the M, T or TL only (without cells present). We did not detect any treatment-associated difference in cell lysate concentrations of TGF-β1.

### TGRL Lipolysis Products Increase Smad2 Phosphorylation

**Fig 2:**
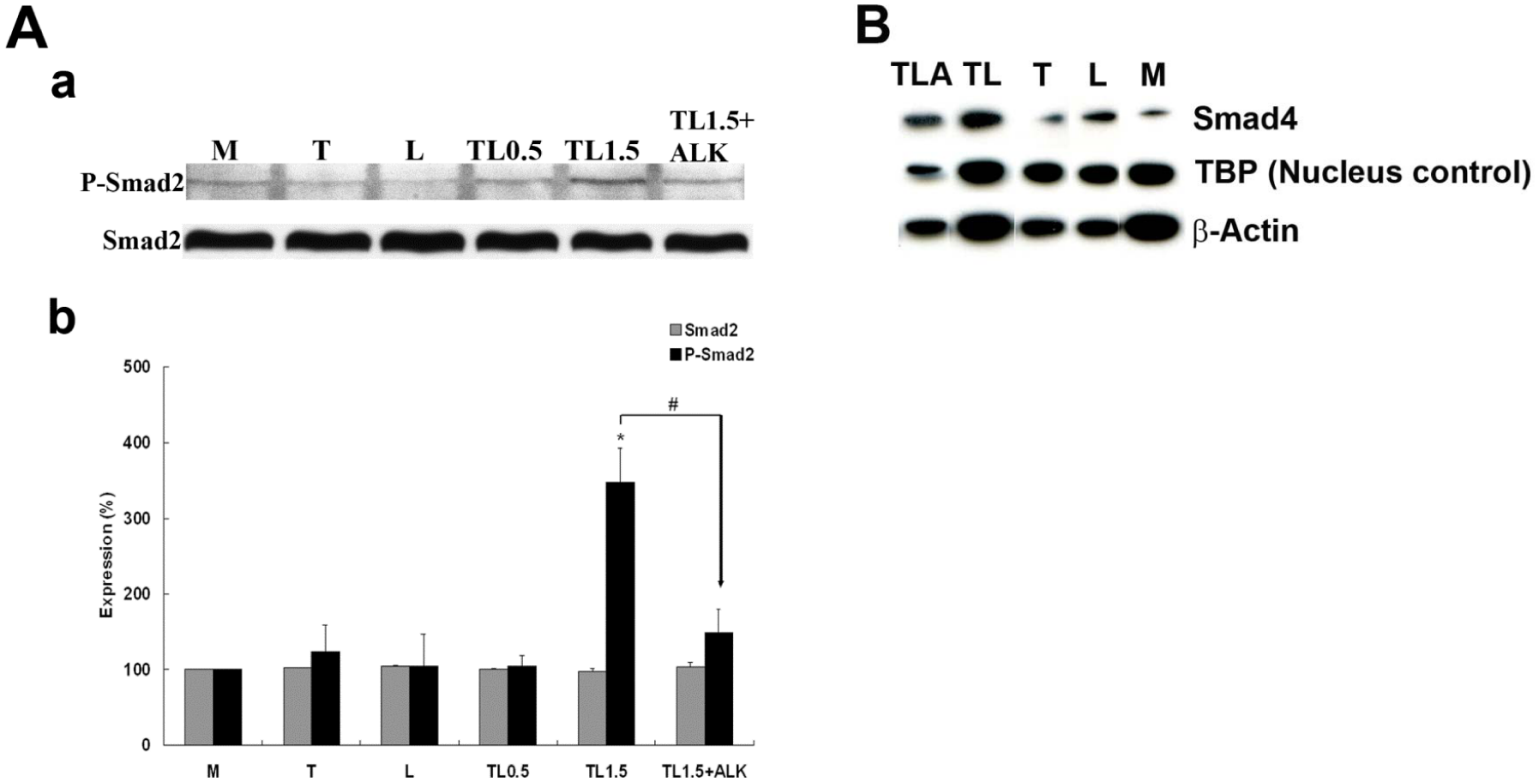
Western Blots of Smad2 phosphorylation at 1.5 h and nuclear expression of Smad4 at 3 h. **A**) Smad2 and Phospho-Smad2 protein. Western Blot (a) and densitometry quantification (b) of Lysates from endothelial cells treated with TGRL (150 mg/dL) + LpL (2 U/mL) TL for 1.5 h (TL1.5) show significantly increased phosphorylation of Smad2 compared to cells treated with media (M), TGRL (T), LpL (L) or TL for 0.5 h (TL0.5). Addition of 10 μM of ALK, TGF-β receptor inhibitor, effectively blocks Smad2 phosphorylation (TL1.5+ALK). N = 3/treatment group, *P*≤0.05 as significant, * = TL1.5 compared to M, L, T, or TL0.5, # = TL1.5+ALK compared to TL1.5. **B)** Smad4 nuclear expression comparing M, L, TL and TLA. Cytosolic fractions were also run on the same blot corresponding to the nuclear fraction. Levels of Smad4 were too low to detect when loaded at a proteins concentration equivalent to the level of protein loaded for the nuclear fraction. These lanes were removed for clarity. Addition of 10 μM of ALK, TGF-β receptor inhibitor, suppressed but did not completely abrogate lipolysis induced Smad4 nuclear accumulation.

**Fig 3:**
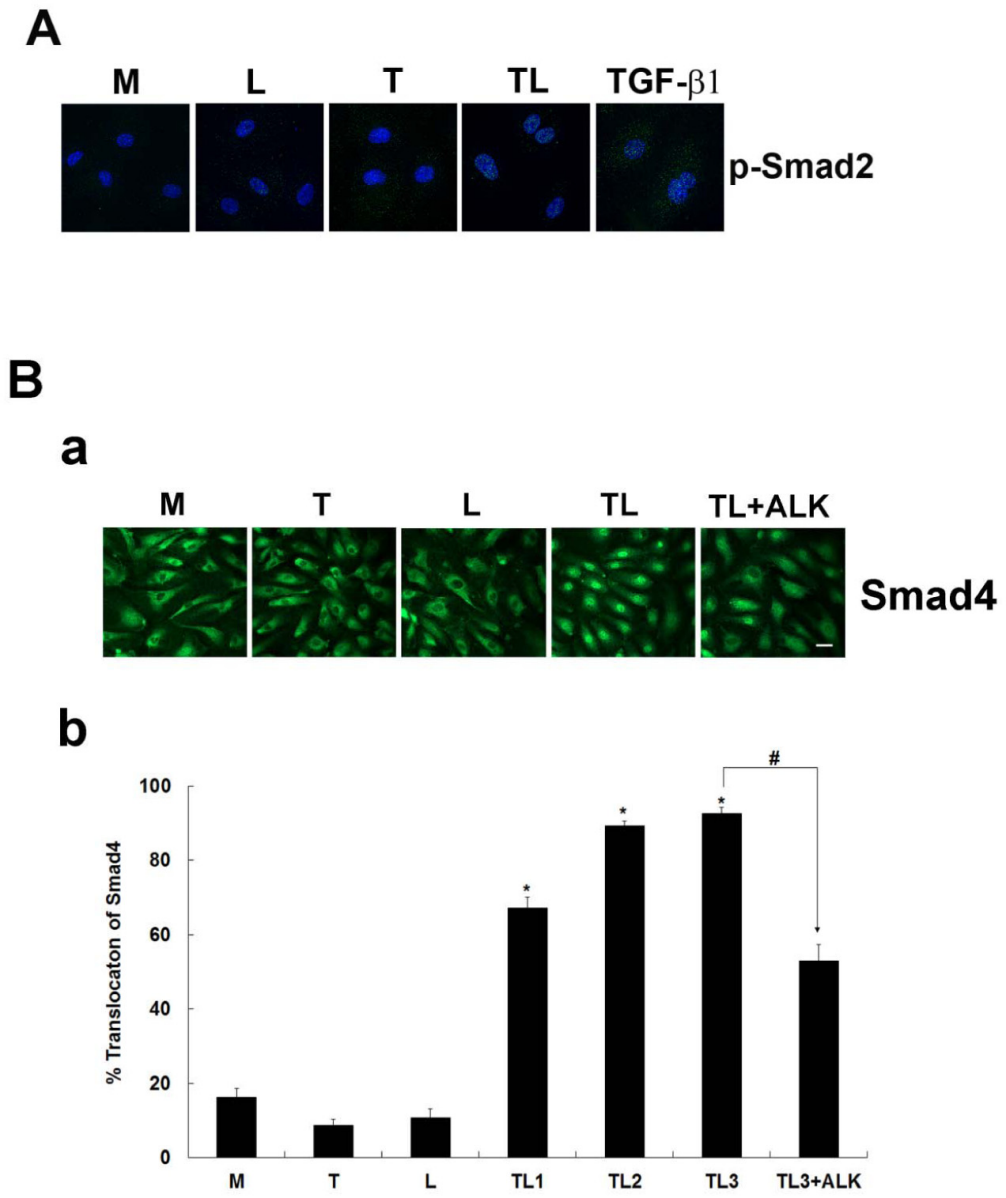
Smad2 phosphorylation at 1.5 h and Smad4 nucleus accumulation at 3 h by lipolysis products. **A**) The nuclear isolated phosphorylated Smad2 was only observed with cellular exposure of lipolysis products (TL) or TGF-β1, not with Media (M), LpL (L) or TGRL (T). **B**) (a) HAEC monolayers show changes in localization of Smad4 from cytosol to nucleus after treatment with lipolysis products compared to controls treated with M, L or T at 1 h. Treatment with lipolysis products with inhibitor ALK (10 μM) shows partial abrogation of Smad4 translocation. (b) % Accumulation of Smad4 based on counts of fluorescent nuclei. Accumulation was significantly increased after 1 h of treatment with lipolysis products (TL1h). The addition 10 μM of inhibitor ALK (TL3 + ALK) significantly reduced the observed accumulation. N = 5 coverslips/treatment group, *P*≤0.05, * = TL1, TL2, TL3 compared to M, # = TL3+ALK compared to TL3 (Bar = 20 μm).

To determine if TGRL lipolysis products could activate TGF-β family second messenger signaling, we determined Smad2 phosphorylation by western blotting, probing for Smad2 as well as p-Smad2 (Ser423/425). While Smad2 protein content was unchanged, we detected a significant increase (~ 3.5-fold) of p-Smad2 in cells treated with lipolysis products at 1.5 h (TL1.5) compared to media control (M) (Fig 2A). Phosphorylation of Smad2 was significantly reduced by the addition of ALK, SB 431542 (10 μM), an inhibitor of the activity of TGF-β1 activin receptor-like kinases (ALKs 4, 5, and 7). Treatment with T, L or 0.5 h of treatment with TGRL lipolysis products (TL0.5) did not increase p-Smad2. Immunofluorescence analysis also showed that phosphorylated Smad2 was in nucleus only following TL exposure (Fig 3A).

### TGRL Lipolysis Products Induce Smad4 Translocation to the Nucleus

To determine whether TGRL lipolysis products could induce Smad4, we determined the Smad4 expression by western bloting, we detected an increase accumulation of Smad4 in the nucleus following exposure to TGRL lipolysis products (Fig 2B). Immunofluorescent labeling also confirmed the increased accumulation of the Smad4 in the nucleus, we characterized localization of Smad4. Our results demonstrated a Smad4 accumulation in the nucleus after treatment with TGRL lipolysis products at 1 h (Fig 3B). While nuclei in control cells treated with M, T or L alone were mostly dark and less green fluorescence, nuclei from cells treated with TL at 1, 2 and 3 h had prominent green fluorescence. Accumulation of Smad4 in the nucleus at 3 h could be partially blocked by the addition of ALK, SB 431542 (10 μM).

Results of cell counts confirmed that significant numbers of cells accumulated Smad4 protein in their nucleus after only 1 h of treatment with lipolysis products, with approximately 67% of cells showing a nuclear localization of Smad4 (TL1h) compared to 16% for the media control (M) (Fig 3B). Smad4 nuclear staining was also observed after 2 and 3 h exposure to lipolysis products (TL2h, TL3h) (data not shown), but these increases were not significantly different from the 1 hour time point. Addition of the ALK, TGF-β receptor inhibitor SB 431542 (10 μM) significantly blocked the accumulation of Smad4 in the nucleus. While the inhibitor did not completely abrogate Smad4 accumulation, nuclear staining was apparent in 53% of inhibitor-treated cells compared with 92% for cells treated with lipolysis products alone for 3 h (Fig 3B).

### TGRL Lipolysis Products Increase p53 Expression

**Fig 4:**
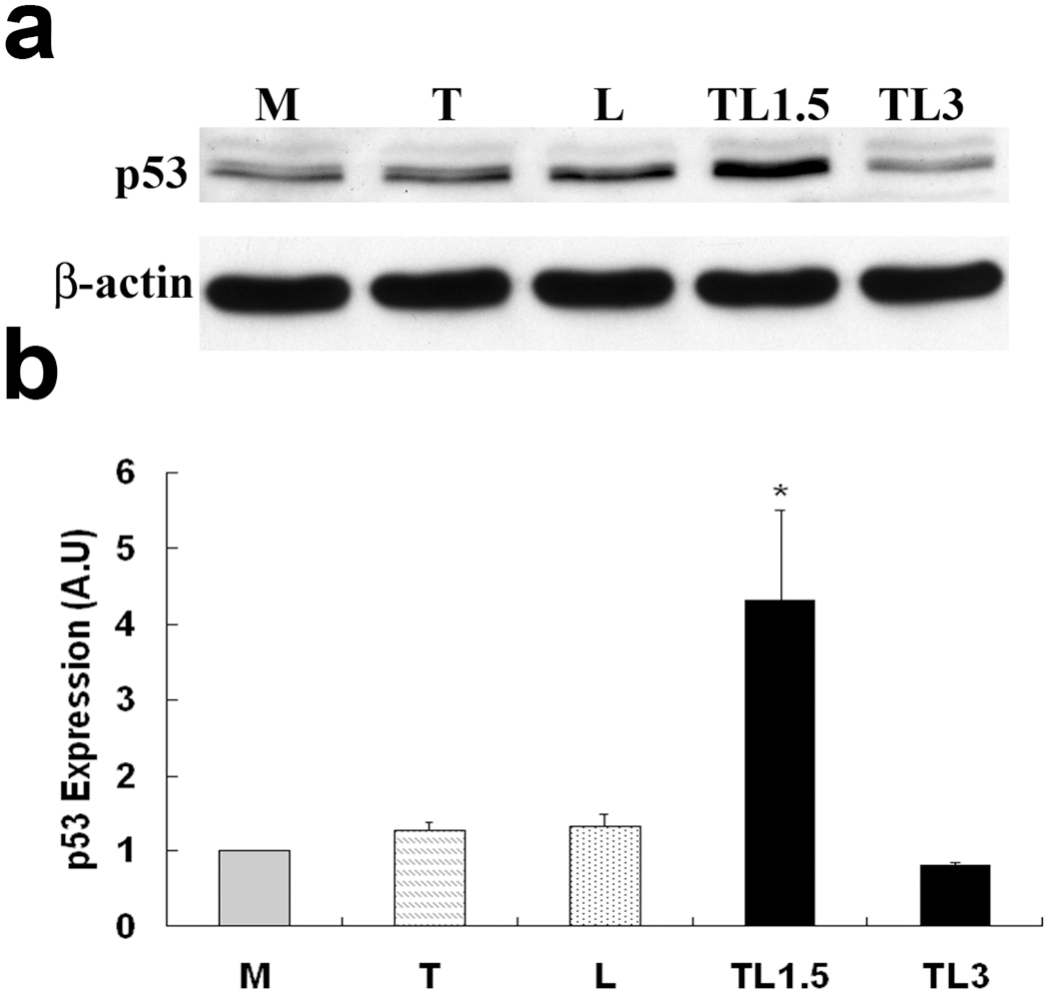
p53 protein expression by TGRL lipolysis products was suppressed by TGF-β receptor inhibitor, ALK. Western Blot (a) and densitometry quantification (b) for p53. p53 expression is significantly increased after 1.5 h of treatment with lipolysis products (TL1.5) compared to control cells (M). Treatment with TGRL (T) or LpL (L) does not alter p53 protein content. N = 3/treatment group, * = *P*≤0.05. Expression declines after 3 h of treatment with lipolysis products (TL3) *P*≤0.1.

To determine if expression of p53, a tumor suppressor protein and regulator of apoptosis was altered in HAEC exposed to lipolysis products, we performed western blots and analyzed optical densities. After 1.5 h of treatment with TGRL lipolysis products (TL1.5), p53 expression was significantly increased (Fig 4), while the treatment with T or L did not significantly alter p53 expression compared to the media control group. Treatment of cells with lipolysis products for 3 h (TL3) resulted in reduced p53 protein expression compared to TL1.5 returning protein content to levels similar to those of the media control. The increase in p53 expression after treatment with lipolysis products (TL1.5) was significantly prevented by addition of ALK, SB 431542 (10 μM) (TL1.5+ALK) in S2 Fig.

To exclude the possibility of cross-reactivity of the antibody to lipolysis products, a control group was included for which TGRL plus LpL was incubated in flasks without cells and then processed and treated similar to other controls. As shown in S2 Fig, no immunoreactivity of the p53 antibody to TGRL lipolysis products was detected without cells (TL-).

### TGRL Lipolysis Products Activate Caspase-3

**Fig 5:**
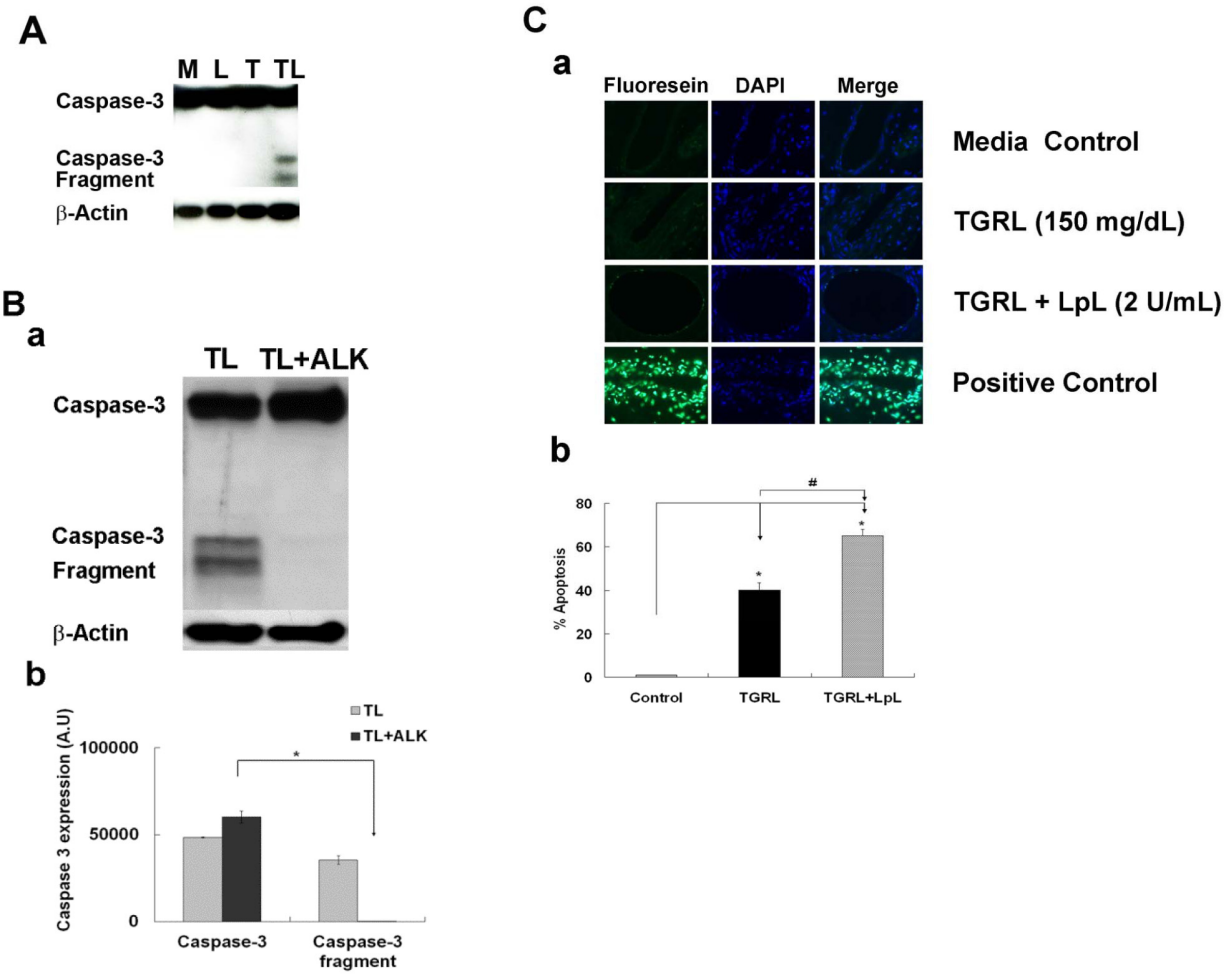
TGRL lipolysis activates apoptosis both *in vitro* and *vivo*. **A)** Lipolysis activates caspase-3 activity in HAEC at 3h. **B)** Caspase-3 protein expression was blocked by TGF-β receptor inhibitor, ALK. Western blots (a) and densitometry quantification (b) for caspase-3. Treatment with lipolysis products increases expression of the active fragment of caspase-3 at 3 h. Additional treatment with 10 μM of inhibitor ALK (TL3+ALK) abrogated the expression of the active caspase-3 fragment. N = 3/treatment group, *P*≤0.05,* = TL compared to TL + ALK. **C)** TGRL lipolysis products activate apoptosis in mouse carotid artery. (a) Terminal deoxynucleotidyl transferase dUTP nick end labeling (TUNEL) staining of mouse carotid artery. (b) Percentage of apoptosis of endothelial cells based on FITC and nuclear staining. TGRL lipolysis (TL) significantly induced apoptosis (65%) compare to control in mouse carotid artery. Positive control: DNAse I treated; negative control: without rTdT enzyme. N = 4 mice/group, *P*≤0.05 as significant, * = T or TL compare to media control group, # = TL compare to T. Original magnification ×60, Bar = 20 μm.

To ascertain whether lipolysis products generated from co-incubation of TGRL with LpL induce the apoptotic cascade, we performed Western Blots for caspase-3 and calculated the expression of its active fragment. We found marked increase in expression of activated caspase-3 after 3 h treatment with lipolysis products (TL3) in Fig 5A and 5B. Treatment with ALK, SB 431542 (10 μM) completely blocked caspase-3 activation.

### TGRL lipolysis products activate apoptosis in mouse carotid arteries

Our data demonstrate that caspase-3 protein expression was increased in *in vitro* HAEC exposed to TGRL lipolysis. To determine whether TGRL lipolysis activates apoptosis in intact arteries, we perfused mouse carotid arteries in situ with Media control, TGRL, or TGRL lipolysis products for 15 min. TUNEL staining showed apoptotic endothelial cells in carotid arteries treated with either TGRL or TGRL lipolysis products. TUNEL staining was not present in carotid artery endothelium from mice perfused with media alone (Fig 5C). Counts of endothelial cells showed carotid arteries perfused with TGRL alone had significantly increased numbers of apoptotic cells (40%) and TRGL lipolysis products further increased the proportion of apoptotic cells (65%) compared to medial alone (Fig 5C).

### Lipolysis Product-Induced Up-regulation of ATF3 is controlled by the TGF-β Receptor

**Fig 6:**
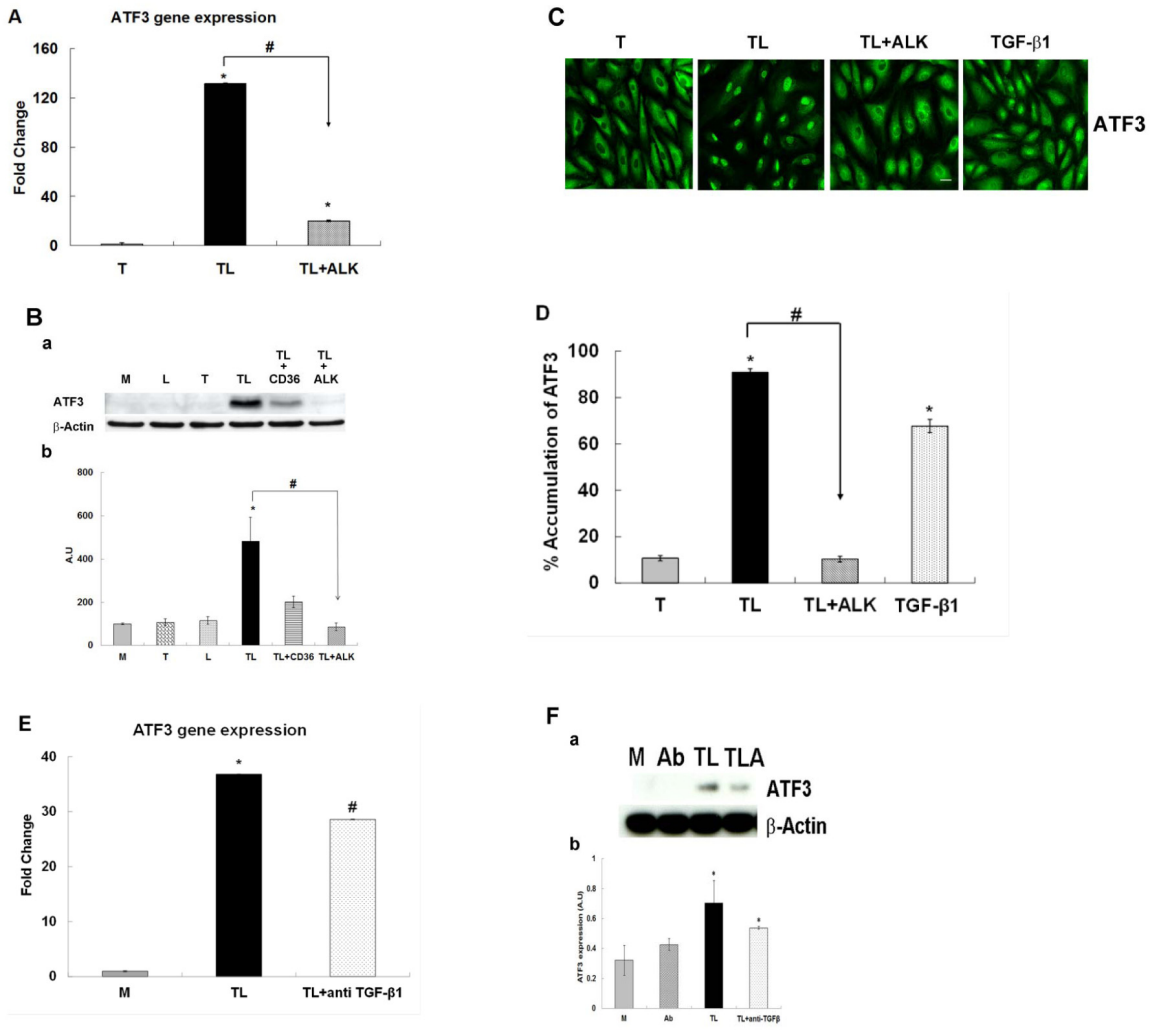
The effect of the ALK 4, 5 and 7 inhibitor or anti TGF-β antibody on the TGRL lipolysis induced ATF3 expression. HAEC were exposed to TGRL (T), TGRL lipolysis products (TL) or 20 ng/mL human TGF-β1 for 3 h. **TGF-β receptor inhibitor, ALK significantly suppressed: A)** mRNA expression of ATF3. N = 3, *P*≤0.05. * = TL compare to T, # = TL with 10 μM of inhibitor ALK (TL+ALK) compared to TL. **B)** Western blot (a) and densitometry quantification (b) for ATF3 protein. N = 3, *P*≤0.05. * = TL compare to T, # = TL+ALK compared to TL. TL+CD36 antibody as control (positive/negative). **C)** Immunofluorescence images showing nucleus accumulation of ATF3. N = 3 coverslips/treatment group, Bar = 20 μm. **D)** % Translocation of ATF3. N = 6 coverslips/treatment group, *P*≤0.05, * = TL or TGF-β1 compare to T, # = TL+ALK compare to TL, Bar = 20 μm. **anti TGF-β1 antibody (Ab) suppressed: E)** mRNA expression of ATF3 was significantly suppressed. N = 3, *P*≤0.05. * = TL compare to M, # = TL+ anti TGF-β1 antibody compared to TL. **F)** Western blot (a) and densitometry quantification (b) ATF3 protein expression was trend toward suppressed significant. N = 3, *P*≤0.05. * = TL or TL + anti TGF-β1 (TLA) compare to M. Ab = anti TGF-β1 antibody.

To assess whether TGRL lipolysis products induced ATF3 is downstream of TGF-β receptor activation, we performed qRT-PCR as well as western blots. Consistent with our previous results [17], we observed a dramatic 130-fold increase in mRNA expression for ATF3 after treatment with TGRL lipolysis products compared to cells treated with TGRL alone (Fig 6A). Addition of 10 μM ALK, SB 431542, an inhibitor to the activity of TGF-βR activin receptor-like kinases (ALKs 4, 5, and 7) (TL + ALK) significantly suppressed (50%) ATF3 mRNA expression compared to treatment with TGRL lipolysis products (TL) at 3 h.

Western blot analysis showed TGRL lipolysis products induced protein expression of ATF3 was also significantly suppressed by addition of 10 μM ALK inhibitor (TL+ALK) (Fig 6B). TGRL lipolysis products increased ATF3 expression significantly at 3 h compared to media, TGRL or LpL alone. The addition of a CD36 blocking antibody to the CD36 lipoprotein receptor was able to partially abolish the observed response of TGRL lipolysis products (Fig 6B).

After treatment with TGRL lipolysis products (TL) for 3 h, ATF3 was shown to be almost exclusively localized in the nucleus with 90% of cells counted as positively stained. Similarly, 70% of cells treated with human TGF-β1 (20 ng/mL) displayed brightly stained nuclei. The addition of the TGF-β receptor inhibitor almost completely abrogated this response (TL+ALK) and only approximately 10% of cells showed nuclear accumulation (Fig 6C and 6D).

Moreover, HAEC treated with TL and anti TGF-β1 antibody (4 μg/mL) also significantly down-regulated mRNA expression of ATF3 (Fig 6D). Protein expression of ATF3 was also suppressed by TL with anti TGF-β1 (Fig 6F).

### ATF3 Binding Partner c-Jun Expression is Regulated by the TGF-β Signaling

**Fig 7:**
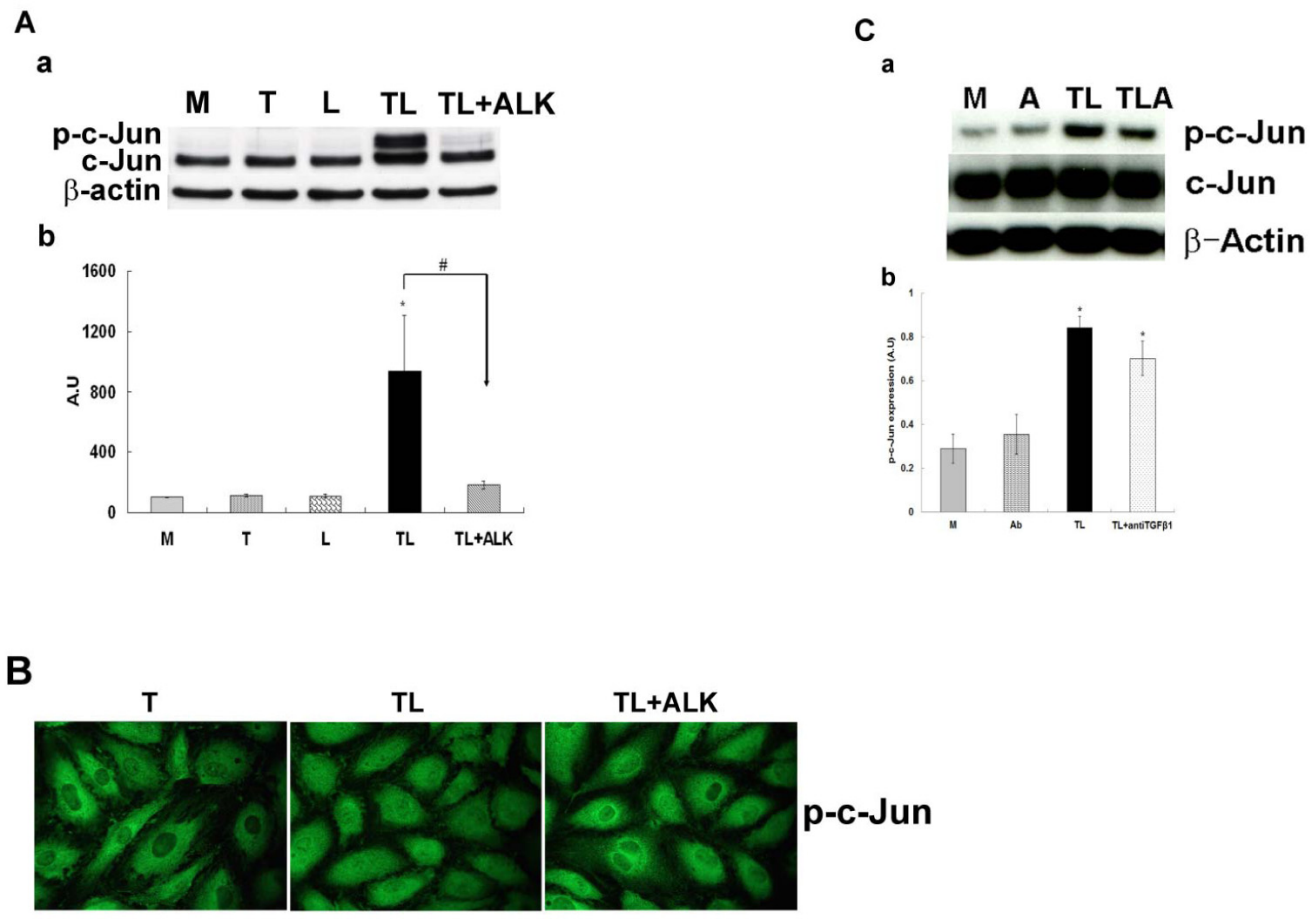
The effect of the ALK 4, 5 and 7 inhibitor or anti TGF-β antibody on the TGRL lipolysis activated phosphorylation of c-Jun protein expression. HAEC were exposed to TGRL (T), TGRL lipolysis products (TL) or TL with 10 μM of inhibitor ALK (TL+ALK) for 3 h. **TGF-β receptor inhibitor, ALK significantly suppressed: A)** Western blot (a) and densitometry quantification (b) for p-c-Jun protein expression. N = 3, *P*≤0.05. * = TL compare to T, # = TL+ALK compared to TL. **B)** Immunofluorescence images showing nuclear translocation of p-c-Jun. N = 3, Bar = 20 μm. **anti TGF-β1 antibody (Ab) suppressed: C)** Western blot (a) and densitometry quantification (b) p-c-Jun protein expression was trend toward suppressed significant. N = 3, *P*≤0.05. * = TL or TL + anti TGF-β1 (TLA) compare to M.

Our previous studies have indicated that TGRL lipolysis products up-regulated expression of transcription factor ATF3 and one of its binding partners c-Jun [17]. To determine whether this induction is downstream of TGF-β receptor activation, we performed western blots on cells treated with media, LpL, TGRL or TGRL lipolysis products (TL) or TL plus ALK, the TGF-β receptor inhibitor, (TL+ALK). While control groups (M, T or L) displayed no changes in c-Jun expression, treatment with TL significantly up-regulated c-Jun and p-c-Jun expression (Fig 7A). When the TGF-β receptor inhibitor was added (TL+ALK), expression levels of c-Jun decreased significantly and p-c-Jun expression was completely blocked by ALK inhibition. The immunofluorescence staining also showed that nuclear accumulation of p-c-Jun was suppressed when the TGF-β receptor inhibitor was added (TL+ALK) (Fig 7B).

Western blot analysis also showed that addition of anti TGF-β1 antibody (4 μg/mL) to TL also suppressed p-c-Jun protein expression (Fig 7C).

### Transcription of E-selectin and IL-8 is Downstream of TGF-β

**Fig 8:**
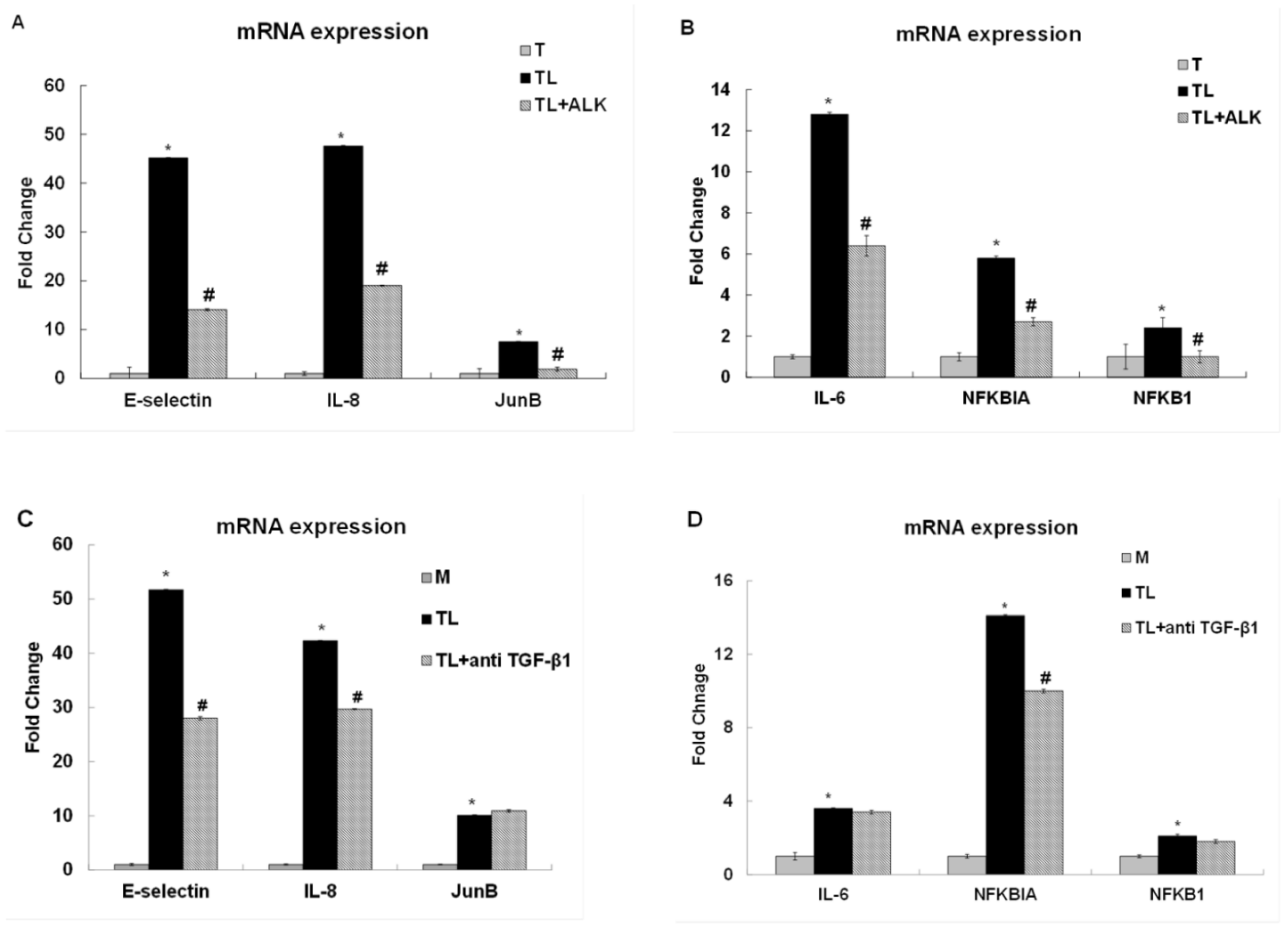
TGF-β receptor inhibitor or anti-TGF-β1 suppressed TGRL lipolysis products-induced pro-inflammatory gene expression. Treatment with lipolysis products increased pro-inflammatory gene expression at 3 h. **Effect of 10 μM of inhibitor ALK (TL+ALK): A)** mRNA expression of E-selectin, IL-8 and JunB expression was significantly suppressed by TGF-β receptor inhibitor. N = 3, *P*≤0.05. * = TL compare to T, # = TL+ALK compared to TL. **B)** mRNA expression of IL-6, NFKBIA/IκBA and NFKB1/NFκB (p50)expression was also significantly suppressed by inhibitor. N = 3, *P*≤0.05. * = TL compare to T, # = TL+ALK compared to TL. **Effect of 4 μg/mL of anti TGF-β1 antibody: C)** mRNA expression of E-selectin and IL-8 expression was significantly suppressed by anti TGF-β1 but not JunB. N = 3, *P*≤0.05. * = TL compare to M, # = TL + anti TGF-β1 compared to TL.

Our previous studies showed that TGRL lipolysis products are involved in the transcriptional regulation of the adhesion molecule E-selectin and the inflammatory cytokines IL-8 and IL-6 [17]. While IL-8 up-regulation was dependent on activation of JNK and ATF3, IL-6 up-regulation was independent of these pathways. To demonstrate that ATF3’s regulatory role of E-selectin and IL-8 is dependent on prior lipolysis product-induced TGF-β receptor activation, HAEC were treated with TGRL, TGRL lipolysis products, or TL+ALK (TGF-β receptor inhibitor) for 3 h. TGF-β receptor inhibitor suppressed 69%, 60% and 75% of TGRL lipolysis products induced E-selectin (45-fold), IL-8 (48-fold) and JunB (7.5-fold) mRNA expression, respectively, compared to TGRL alone (Fig 8A). TGRL lipolysis product-induced up-regulation of the inflammatory cytokine IL-6 (12.8-fold), NFKBIA (5.8-fold) and NFKB1 (2.4-fold) were suppressed 50%, 47% and 42% by TGF-β1 receptor inhibitor (Fig 8B).

Additionally, anti TGF-β1 antibody also suppressed TGRL lipolysis products-induced E-selectin (46%) and IL-8 (30%) gene expression but not JunB (Fig 8C). The expression of NFKBIA was suppressed 29% although IL-6 and NFKB1 were not suppressed by anti TGF-β1 antibody (Fig 8D).

## Discussion

We have demonstrated that treatment with high-physiological levels of lipolysis products generated from TGRL isolated from healthy human volunteers, led to TGF-β secretion from cultured endothelial cells and subsequent autocrine TGF-β receptor activation, which, as far as we know, is a novel observation. However, there has been significant controversy over TGF-β’s role in the development of atherosclerosis [41, 42]. This cytokine displays a wide range of actions in all cell types that are known to be present in atherosclerotic lesions. Due to the array of responses that TGF-β1 can elicit in different cell types, but also in the same cell type depending on serum levels, concentration, expression level of receptors and stage in the cell cycle, it has been difficult to put a distinctive label on this multi-functional cytokine. While initially presumed to be pro-atherogenic, since it was found at increased levels in vascular lesions [10, 43] and in aged aorta [44] and was shown to induce endothelial barrier dysfunction [45–47], additional studies have suggested that TGF-β is also anti-inflammatory and therefore anti-atherogenic. In endothelial cells specifically, TGF-β has been shown to inhibit cytokine-induced expression of adhesion proteins [48] as well as chemoattractants [49–51]. In the present study, we show that TGF-β1 released from HAEC cultures in response to treatment with lipolysis products can activate signaling cascades that lead to endothelial cell apoptosis. This finding is consistent with previous studies demonstrating TGF-β-induced endothelial cell death [24, 52]. While cell turnover occurs normally in healthy vessels, deranged or increased apoptosis of the endothelium has been associated with the development of atherosclerotic cardiovascular disease [1, 53]. This is thought to occur due to 1) an increased pro-coagulant state during endothelial apoptosis [54] and 2) the formation of membrane vesicles and blebs that contain biologically active oxidized phospholipids, which can induce monocyte adhesion [55]. This study as well as our previous findings [13, 17] suggests lipolysis products induce endothelial cell apoptosis independent from leukocytes or circulating proteins in blood.

Previous studies have demonstrated that modified lipoproteins, such as oxidized LDL, can stimulate TGF-β/Smad signaling and apoptosis in different cell types [56, 57] and that lipolysis of TGRL can generate oxylipids such as 13-HODE, the most prevalent oxylipid in oxidized LDL [58]. We have shown that lipolysis products can similarly activate intracellular signaling cascades. Our findings indicate that this occurs through TGRL lipolysis product-mediated release of TGF-β1 and autocrine activation of receptors. We specifically report that the observed increase in TGF-β1 concentration in HAEC supernatants after treatment with lipolysis products translates into the activation of TGF-β membrane receptors and Smad proteins. Upon ligand binding, type I (activin receptor-like kinases, ALKs) and type II TGF-β receptors form heteromeric complexes which facilitate the transphosphorylation of the type I receptor by the type II receptor. In most cells, TGF-β1 signals via TβRII and TβRI (also termed ALK5) to induce phosphorylation of Smad2. Our study demonstrates that lipolysis product-mediated release of TGF-β1 specifically activates TGF-β receptors leading to Smad2 phosphorylation. We also show that an inhibitor of the ALKs 4, 5, and 7 TGF-β type I receptor subtypes almost completely abrogates the induced phosphorylation of Smad2 and, blocks the translocation of Smad4 to the nucleus. A possible explanation of the partial attenuation of Smad4 translocation could be signaling via the endothelial-restricted type I receptor, ALK1 [59]. This receptor, in contrast to the ALK5 receptor, activates Smad1. Activated Smad1 also forms complexes with Smad4 with subsequent translocation to the nucleus.

Hyman et al. [24] showed that TGF-β causes apoptosis by stimulating the mitogen-activated protein kinase (MAPK) p38 in human umbilical vein or bovine capillary endothelial cells. Recent publications demonstrate that TGF-β1 can also trigger apoptosis via the mitochondrial pathway and the tumor suppressor protein p53. These studies showed that p53 expression is up-regulated in various cell types after treatment with TGF-β1 [30, 48, 57, 60]. p53 activation can be induced by a number of stressors, including DNA damage [52, 61], oxidative stress [62, 63], osmotic shock [64], and cytokines [65–67]. Activation of p53 is characterized by two major events: 1) a marked increase of the half-life of p53 protein by escape from proteasome-dependent degradation, which leads to a quick accumulation of the protein and 2) the conversion of the conformation of the DNA-binding domain into a form with high affinity for target DNA, which allows p53 to play an active role as a transcription regulator [68]. In our study we were able to detect increased protein levels of p53 after 1.5 hours of treatment with lipolysis products and were able to demonstrate that the observed p53 induction was dependent upon TGF-β ligand/receptor interaction. The exact mechanisms whereby the activity of growth factors such as TGF-β1 intersect with p53 signaling is still not well understood. However, it is now established that p53 can physically interact with Smad2/3 to jointly regulate the transcription of several TGF-β target genes [28, 69, 70]. In contrast, other studies seem to indicate that TGF-β1 and p53 mediate distinctive apoptotic pathways [71, 72] due to different time courses of activity. Interestingly, p53 up-regulation was evident in earlier time points but not at 3 h post treatment correlating with short term TGF-β expression and suggesting downstream adaptation responses. Our findings demonstrate that for lipolysis product-induced apoptosis in HAEC cultures, activation of TGF-β receptors is required for p53 up-regulation and caspase-3 activation.

Our recent study showed the stress-inducible protein ATF3 [17], was impressively up-regulated in response to lipolysis products. Increasing evidence links this protein to the TGF-β signaling system [39] and also the regulation of endothelial cell apoptosis [21]. The present study was designed to determine, whether TGRL lipolysis products induce ATF3 via the TGF-β1 signaling cascade.

Our data demonstrate that the lipolysis product-induced up-regulation of ATF3, both at the mRNA and the protein level, is dependent on prior activation of the TGF-β receptor. While treatment with lipolysis products alone elicited a strong and impressive up-regulation of ATF3, pre-treatment with an inhibitor to the activity of TGF-β1 activin receptor-like kinases (ALKs 4, 5, and 7) and anti-TGF-β antibody remarkably inhibited this response both at the mRNA and trended in this direction relative to protein level. ATF3 protein expression was partially inhibited by CD36 receptor blocking antibody. CD36 is a glycoprotein and binds to collagen, thrombospondin, anionic phospholipids and oxidized LDL contribute to important pathological processes highly relevant to atherosclerosis and thrombosis [73]. This finding data suggests that CD36 may recognize and bind specific oxidized phospholipid derived from TGRL lipolysis products that CD36 may participate in initiating the ATF3 related stress response. The interplay between and possible regulatory role of TGF-β and ATF3 is still not clear. Both proteins have been previously demonstrated to be potent inducers of endothelial cell apoptosis, were found to be highly expressed at sites of vascular lesions [21], and are both up-regulated in response to pathogenic stimuli like oxidized low density lipoprotein [21, 35, 56] or TNFα [74, 75]. In epithelial cells, ATF3 mRNA as well as protein levels showed an immediate and lasting induction in response to TGF-β [39]. The up-regulated expression was linked to binding of activated Smad2/3 and Smad4 proteins on a TGF-β-responsive region of the ATF3 promoter. A recent report by Jin [76] suggests that in mouse testis TGF-β activates both Smad signaling and non-Smad signaling pathways (MAPK). The authors suggest that the activated MAPK would then contribute to the up-regulation of transcription factors, such as ATF3 or c-Jun to regulate the expression of anti-oxidant enzymes. Since our previous work has linked lipolysis product treatment of endothelial cells to an increased generation of reactive oxygen species, it will be of future interest to pursue the notion that this phenomenon could be linked to prior TGF-β receptor activation/up-regulation of ATF3 and could possibly be blocked by the addition of TGF-β receptor inhibitors.

In addition to the regulation and suppression of antioxidant enzymes, ATF3 has also been implicated in the control of several other genes associated with the development of atherosclerosis, such as the expression of the adhesion molecule E-selectin and the cytokine IL-8 by our group [17] and others [77, 78]. While the present study clearly shows that lipolysis products up-regulate those genes and this increase in expression depends on prior TGF-β receptor activation, another study reports that, at least in the case of E-selectin, ATF3 acts as a transcriptional repressor, not activator [77]. This difference in results might stem from the fact that ATF3 can form homodimers as well as heterodimeric complexes with other transcription factors, such as c-Jun, ATF2, gadd153/CHOP10, JunD and JunB [79] and thereby mediate different responses, depending on stimulus, cell type, and cell context.

As a transcription factor, ATF3’s activity depends on its ability to reach DNA binding sites and accumulate to the nucleus. Our immunofluorescence studies show that during treatment with lipolysis products, ATF3 quickly accumulates in the nucleus. A similar response was observed when cells were treated with TGF-β directly. Pre-treatment of the endothelial cell layer with an inhibitor of the TGF-β receptor lead to significant blockage of ATF3 nuclear localization. Previous reports have linked ATF3 up-regulation to a preceding or concurrent induction and phosphorylation of the transcription factor c-Jun and have shown that the increase in ATF3 expression can be blocked by knockdown of c-Jun [80–82]. While previous reports have demonstrated that ATF3 homodimeric complexes act as repressors for gene transcription, heterodimeric ATF3/c-Jun complexes seem to act as activators for gene transcription [78, 83]. Additionally, known risk factors for the development of atherosclerotic cardiovascular disease can induce ATF3 in a c-Jun–dependent manner. Cai et al. (2000) and Zhang et al (2001), for example, demonstrate that homocysteine leads to a rapid and sustained induction of c-Jun and precedes the induction of ATF3 [84, 85]. Here, we show that treatment of HAEC with TGRL lipolysis products leads to increased phosphorylation of c-Jun, which occurred concurrently with our observed up-regulation of ATF3. It remains to be tested whether there is a physical interaction between ATF3 and c-Jun to form previously reported heterodimeric complexes [78, 83], that may act as transcription factors to regulate the transcription of target genes.

The lipolysis product-induced increase in c-Jun expression was in a manner similar to ATF3, blocked by the addition of an inhibitor of the TGF-β receptor or anti-TGF-β antibody. This suggests that both c-Jun and ATF3 are under the regulatory control of the TGF-β/Smad signaling cascade. A study by Jin et al. also suggests a link between Smad signaling and c-Jun/ATF3 and shows that silencing Smad2 with siRNA partially attenuates expression of c-Jun and ATF3 [76]. The direct effects of Smad2 on c-Jun and ATF3 have not been investigated.

In our previous study [17] we found ATF3 expression was necessary for induction of some, but not all, pro-inflammatory responses to lipolysis product treatment. In the present study inhibition of the TGF-β receptor (ALK) suppressed pro-inflammatory gene expression related to AP-1 signaling (E-selectin, IL-8) and NFκB signaling (IL-6, NFKB1, NFKBIA) by TGRL lipolysis products. In contrast, findings including lack of IL-6, NFKB1 (NFκB), and JunB responsiveness and decreased NFKB1 (IκBA) by anti-TGF-β antibody suggest that anti-TGF-β antibody only inhibited JNK pathway but not NFκB signaling in response to lipolysis products. Both the antibody blocking of TGF-β and the use of the ALK receptor inhibitor lend support to the involvement to a TGF-β signaling pathway in the cellular response to lipolysis products.

To determine the relevance of our *in vitro* findings to intact arteries, we performed a similar experiment by perfusing mouse arteries in situ. Our findings demonstrate the induction of apoptosis by lipolysis products in intact arteries. In addition, perfusion of TGRL alone induced a significant percentage of apoptotic cells suggesting that LpL expressed in endothelium from intact arteries is sufficient to elicit lipolysis product induced signaling.

**Fig 9:**
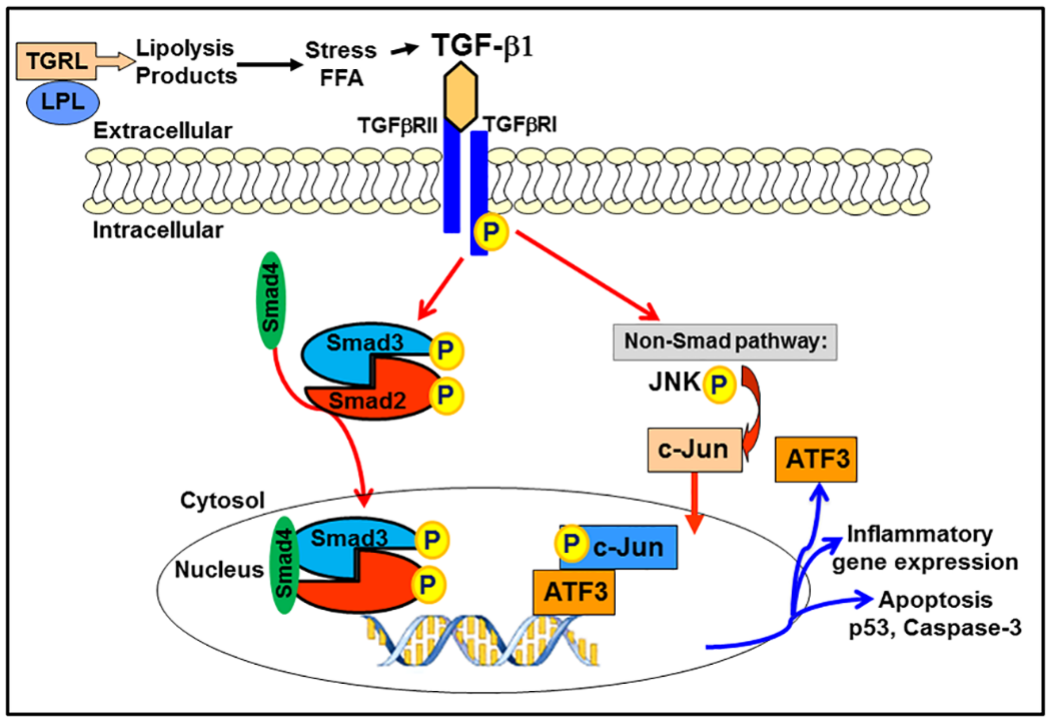
TGRL lipolysis products activate stress response signaling via TGF-β/SMAD Signaling Pathway. Lipolysis release TGF-β1 and activate phosphorylation of Smad2 and translocation of Smad4 to nucleus. TG-β1 also activate non-Smad signaling pathways ATF3-JNK transcription factor networks. Both Smad and ATF3 further induced pro-inflammatory cytokines and apoptosis which can be inhibited by TGF-β receptor inhibitor, ALK.

In summary, we have made novel observations that TGRL lipolysis products stimulate release of active TGF-β1 and autocrine activation of the Smad signaling cascade in endothelial cells. We confirm that lipolysis of postprandial lipids induces the up-regulation of the stress protein ATF3 in human aortic endothelial cells and this up-regulation is under the regulatory control of the TGF-β/Smad signaling cascade both at the mRNA as well as the protein level. Our study demonstrates that ATF3 mediatedTGRL lipolysis induced inflammation and apoptosis is dependent on the TGF-β/Smad signaling pathway (Fig 9). While the exact interplay between the ATF3 and TGF-β signaling network is still largely unknown and represents a fertile area for further investigation, the present study points to the TGF-β receptor as a potential target for intervention to block the up-regulation of stress proteins, like ATF3, a master regulator of endothelial cell inflammatory responses.

## Supporting Information

S1 Fig TGRL lipolysis products release TGF-β1. **A**) TGF-β1 release at 30 min. **B**) TGF-β1 release at 45 min. The rate of TGF-β1 release is no changed for cells treated with with Media (M) or LpL alone (L) or TGRL alone (T), TGRL (150 mg/dL) + LpL (2 U/mL) (TL) or addition of 10 μM of ALK to TL (TL+ALK), at 30 min or 45 min. N = 4/treatment group.
(TIF)

S2 Fig p53 protein expression by TGRL lipolysis products was suppressed by TGF-β receptor inhibitor, ALK. Western Blot (a) and densitometry quantification (b) for p53. The increase in p53 expression after treatment with lipolysis products (TL1.5) was prevented by addition of ALK4, 5 and 7 inhibitor (TL1.5+ALK). No immunoreactivity of lipolysis products only was detected to the p53 antibody (TL-). Decreased p53 expression in response to lipolysis products in TL1.5+ALK cells was statistically significant. N = 3/treatment group, * = *P*≤0.05.
(TIF)

S1 ARRIVE Checklist (PDF)
