# Supplementary figures and images for: TGRL Lipolysis Products Induce Stress Protein ATF3 via the TGF-β Receptor Pathway in Human Aortic Endothelial Cells
Source: PLoS One. 2015 Dec 28;10(12):e0145523. doi: 10.1371/journal.pone.0145523 (PMC4699200; doi:10.1371/journal.pone.0145523)

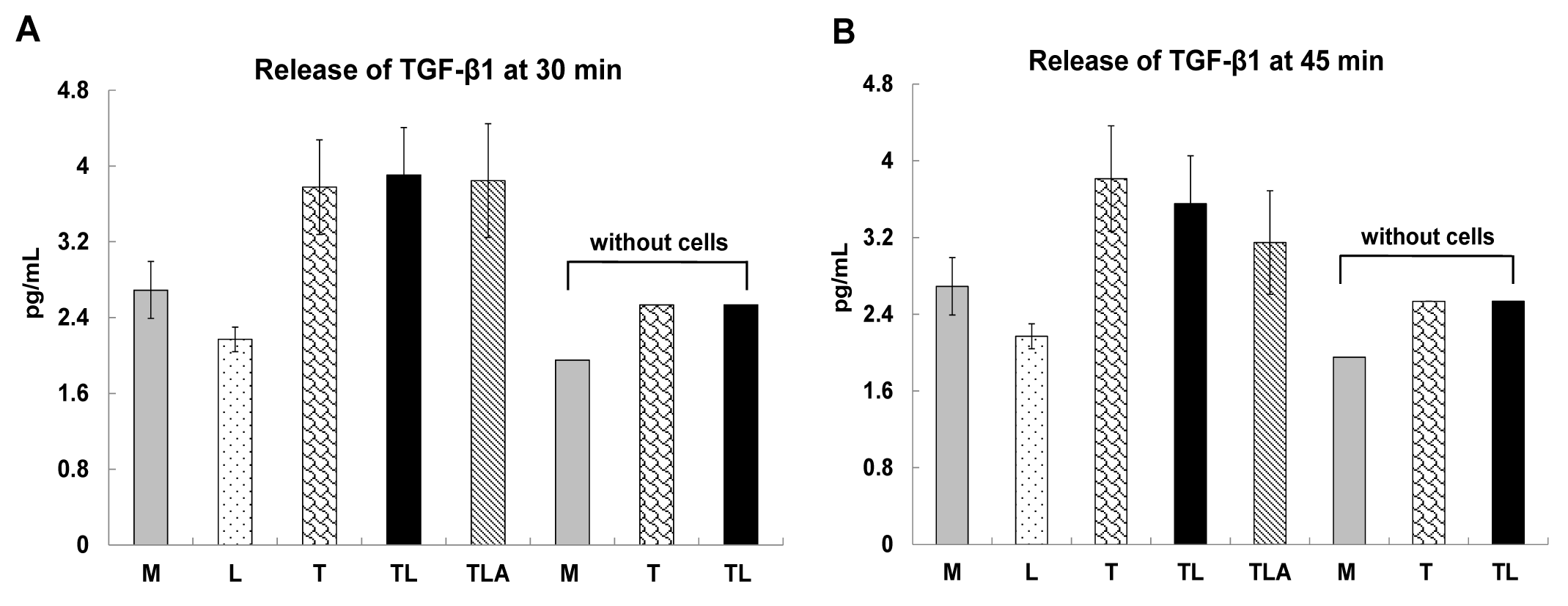

Supplement: S1 Fig — A) TGF-β1 release at 30 min. B) TGF-β1 release at 45 min. The rate of TGF-β1 release is no changed for cells treated with with Media (M) or LpL alone (L) or TGRL alone (T), TGRL (150 mg/dL) + LpL (2 U/mL) (TL) or addition of 10 μM of ALK to TL (TL+ALK), at 30 min or 45 min. N = 4/treatment group. (TIF) [file pone.0145523.s001.tif]

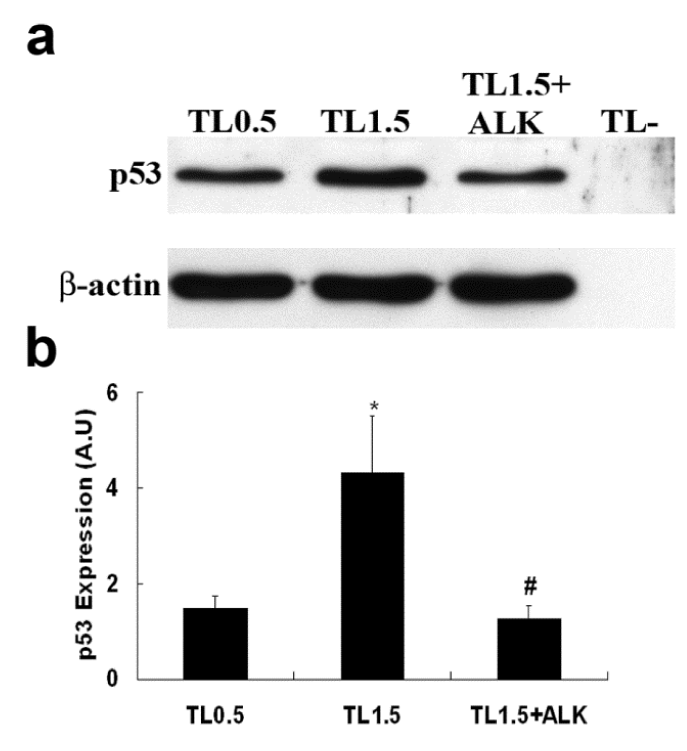

Supplement: S2 Fig — Western Blot (a) and densitometry quantification (b) for p53. The increase in p53 expression after treatment with lipolysis products (TL1.5) was prevented by addition of ALK4, 5 and 7 inhibitor (TL1.5+ALK). No immunoreactivity of lipolysis products only was detected to the p53 antibody (TL-). Decreased p53 expression in response to lipolysis products in TL1.5+ALK cells was statistically significant. N = 3/treatment group, * = P≤0.05. (TIF) [file pone.0145523.s002.tif]
